# Over-expression of mitochondrial creatine kinase in the murine heart improves functional recovery and protects against injury following ischaemia–reperfusion

**DOI:** 10.1093/cvr/cvy054

**Published:** 2018-03-02

**Authors:** Hannah J Whittington, Philip J Ostrowski, Debra J McAndrew, Fang Cao, Andrew Shaw, Thomas R Eykyn, Hannah A Lake, Jack Tyler, Jurgen E Schneider, Stefan Neubauer, Sevasti Zervou, Craig A Lygate

**Affiliations:** 1 Division of Cardiovascular Medicine, Radcliffe Department of Medicine, The Wellcome Centre for Human Genetics, and the BHF Centre of Research Excellence, University of Oxford, Roosevelt Drive, Oxford OX3 7BN, UK; 2 School of Biomedical Engineering and Imaging Sciences, King’s College London, King’s Health Partners, St Thomas’ Hospital, London, UK; 3 Experimental and Preclinical Imaging Centre (ePIC), Leeds Institute of Cardiovascular and Metabolic Medicine, University of Leeds, Leeds, UK

**Keywords:** Cardiac energetics, Metabolism, Creatine kinase, Reperfusion injury, Myocardial infarction

## Abstract

**Aims:**

Mitochondrial creatine kinase (MtCK) couples ATP production via oxidative phosphorylation to phosphocreatine in the cytosol, which acts as a mobile energy store available for regeneration of ATP at times of high demand. We hypothesized that elevating MtCK would be beneficial in ischaemia–reperfusion (I/R) injury.

**Methods and results:**

Mice were created over-expressing the sarcomeric MtCK gene with αMHC promoter at the *Rosa26* locus (MtCK-OE) and compared with wild-type (WT) littermates. MtCK activity was 27% higher than WT, with no change in other CK isoenzymes or creatine levels. Electron microscopy confirmed normal mitochondrial cell density and mitochondrial localization of transgenic protein. Respiration in isolated mitochondria was unaltered and metabolomic analysis by ^1 ^H-NMR suggests that cellular metabolism was not grossly affected by transgene expression. There were no significant differences in cardiac structure or function under baseline conditions by cine-MRI or LV haemodynamics. In Langendorff-perfused hearts subjected to 20 min ischaemia and 30 min reperfusion, MtCK-OE exhibited less ischaemic contracture, and improved functional recovery (Rate pressure product 58% above WT; *P* < 0.001). These hearts had reduced myocardial infarct size, which was confirmed *in vivo*: 55 ± 4% in WT vs. 29 ± 4% in MtCK-OE; *P* < 0.0001). Isolated cardiomyocytes from MtCK-OE hearts exhibited delayed opening of the mitochondrial permeability transition pore (mPTP) compared to WT, which was confirmed by reduced mitochondrial swelling in response to calcium. There was no detectable change in the structural integrity of the mitochondrial membrane.

**Conclusions:**

Modest elevation of MtCK activity in the heart does not adversely affect cellular metabolism, mitochondrial or *in vivo* cardiac function, but modifies mPTP opening to protect against I/R injury and improve functional recovery. Our findings support MtCK as a prime therapeutic target in myocardial ischaemia.

## 1. Introduction

The supply of cellular energy in the form of ATP is closely matched to demand, yet ATP levels do not change in the normal heart even when workload is increased suddenly. In the short-term, this is due to the creatine kinase (CK) phosphagen system, which acts to buffer ATP levels and signals for increased energy production.^1^ When demand is sustained, this bridges the gap until rising levels of free calcium activate mitochondrial dehydrogenases to stimulate *de novo* ATP synthesis.^2^

The mitochondrial isoform of CK (MtCK) catalyses the transfer of a phosphoryl group from ATP onto creatine to form phosphocreatine (PCr) and ADP. Phosphocreatine is smaller and less polar than ATP, and is therefore more readily diffusible and accumulates to higher levels within the cell.^3^ It can be rapidly converted back to ATP under control of cytosolic CK enzymes (Muscle and Brain isoforms, which are largely bound to intracellular structures^4–6^) at times of high energy demand or when energy supply is impaired, e.g. at the onset of ischaemia.^7^ The CK system negates the need for ATP and ADP diffusion, which allows fine control of the ATP/ADP ratio in different cellular compartments, thereby optimizing reaction conditions and maintaining a high free-energy of ATP hydrolysis.^3^^,^^7^^,^^8^

Striated muscles, such as heart, express ‘sarcomeric’ MtCK, with the so-called ‘ubiquitous’ MtCK isoenzyme performing the equivalent role in most other tissues.^9^ Under normal conditions, MtCK forms octamers that exactly span the mitochondrial inter-membrane space. At the outer membrane, Mt-CK forms a complex with the voltage-dependent anion channel (VDAC), which regulates creatine/PCr exchange, and at the inner membrane interacts with cardiolipin to allow close coupling with the adenine nucleotide translocase (ANT), which regulates ADP/ATP exchange.^9–11^ This functional unit links cytosolic energy requirements with ATP production via oxidative phosphorylation,^12^ and furthermore, these contact points also play a structural role to stabilize the mitochondrial membrane.^10^

Ischaemia represents an acute crisis in cellular energy provision, but the challenge to cell survival also comes during reperfusion, when high intracellular calcium, rising pH and generation of reactive oxygen species (ROS) all act to stimulate opening of the mitochondrial permeability transition pore (mPTP), which ultimately leads to cell death. This is termed ischaemia/reperfusion (I/R) injury and represents a key therapeutic target to minimize myocardial damage following revascularization procedures.^13^ MtCK is of particular interest in I/R injury since creatine has been shown to reduce mPTP opening probability,^14^^,^^15^ but only when MtCK is localized to the inter-mitochondrial membrane.^14^ Over-expression of MtCK in murine liver has been shown to prevent mPTP opening in response to calcium overload,^16^ and we have recently demonstrated that MtCK over-expression *in vitro* protects against hypoxia/reoxygenation induced cell death.^17^ Furthermore, double CK knockout mice (mito- and M-CK) exhibit increased accumulation of calcium during ischaemia and greater susceptibility to I/R injury,^18^ and MtCK activity closely correlates with recovery of contractile function in post-ischaemic myocardium.^19^

We therefore hypothesized that over-expression of MtCK would be beneficial in the setting of I/R. Here, we describe a novel mouse model of cardiac MtCK over-expression (MtCK-OE), with mitochondrial localization and elevated MtCK activity. This was well tolerated with no effect on mitochondrial cell density, respiration or *in vivo* cardiac function. We show that MtCK-OE hearts have improved functional recovery and reduced myocardial injury following I/R, and that isolated cardiomyocytes and mitochondria are resistant to mPTP opening.

## 2. Methods

Detailed methods can be found in the Supplementary material online.

### 2.1 Animal husbandry

This investigation was approved by the University of Oxford Animal Welfare and Ethical Review Board and conforms to the Animals (Scientific Procedures) Act 1986 incorporating Directive 2010/63/EU of the European Parliament. Mice were kept in specific pathogen-free cages, 12-h light–dark cycle, controlled temperature and humidity, and had water and food *ad libitum* (Teklad global 16% rodent diet). All animals were generated by hemizygous cross and compared to age and sex matched littermates.

### 2.2 Generation and breeding of transgenic mice

Mouse sarcomeric *Ckmt2* with an HA-tag was cloned into a vector containing the αMHC promoter and the machinery for PhiC31 integrase mediated cassette exchange at the *Rosa26* locus. The exchange vector was co-electroporated with an expression cassette for PhiC31 into acceptor embryonic stem cells. Transgenic mice were generated by microinjecting embryonic stem cells into C57BL/6 J blastocysts, which were implanted into pseudo-pregnant females to create chimeric founder mice. Male chimeric founders were mated with wild-type C57BL/6 J females (Envigo, Huntingdon, UK). Homozygous transgenic (Tg^+/+^) mice were generated by breeding F1 Tg^+/–^ males with F1 Tg^+/–^ females and back-crossed onto C57BL/6 J genetic background for 10 generations. Transgenic pups were identified by PCR from ear biopsies as detailed in the Supplementary material online.

### 2.3 Tissue harvest and biochemistry

Mice were killed by cervical dislocation, tissue harvested, washed and blotted in saline, snap frozen in liquid nitrogen, and stored at –80°C until used. Creatine was quantified by HPLC and then normalized to protein content using the Lowry method in *n* = 10 left ventricular (LV) samples per genotype (aged 9 weeks, 50% male). Citrate synthase activity was measured spectrophotometrically in the same samples. Total CK activity was measured in LV homogenates spectrophotometrically via a coupled-enzyme assay in 12 week old mice (*n* = 25 WT, *n* = 18 Tg^+/^^-^, *n* = 15 Tg^+/+^). Using the same samples, individual CK isoenzymes were separated by electrophoretic mobility on an agarose gel and a coupled-enzyme assay performed *in situ* using the SAS-1 CK VIS-12 Isoenzyme kit (Helena Biosciences). Absolute activities for each isoenzyme were calculated by multiplying relative isoenzyme activity (measured by densitometry) with total CK activity. For octamer/dimer visualization, isolated mitochondrial were pre-incubated with and without transition site analogue complex (TSAC) to promote formation of dimers.^20^ We have previously verified the identity of MtCK bands using MtCK knockout tissue and CK inhibitors.^17^

### 2.4 ^1^H NMR metabolomics

Crushed frozen heart samples from 14 week old female mice underwent a standard dual-phase extraction protocol to separate aqueous and lipid layers (*n* = 6 WT; *n* = 6 Tg). Samples were analysed on a Bruker 9.4 T (400 MHz) spectrometer with bbo probe at 298 K. Detailed sample preparation and scan parameters are included in the Supplementary material online. TopSpin (version 2.1) software was used for data acquisition and for metabolite quantification. Peak areas were normalized to the internal standard peaks and metabolite concentrations quantified per gram tissue wet weight. Data is shown as a fold change in mean metabolite concentrations relative to wild-type control samples with a propagated standard error (SEM) of the ratio. Unsupervised Principle Component Analysis was performed in Matlab.^21^ Between group comparison was by Student’s *t*-test.

### 2.5 Electron microscopy

Hearts from male WT and MtCK-OE mice (30 weeks of age) were cannulated via the aorta, perfusion fixed, and prepared as described in Supplementary material online. Sections were incubated with rat anti-HA primary antibody, followed by an anti-rat conjugated to 5 nm gold, before post-staining with uranyl acetate and Reynold’s lead citrate, and imaging using a FEI Tecnai 12 TEM at 120 kV. Similar tissue preparation with slight modifications was used to assess mitochondrial cell density in *n* = 6 mouse hearts. Samples were blinded to the operator and 18–20 images were taken per heart at two magnifications, 890x, and 1900x, within organized Z-line sections. Images were analysed in Image J (v1.50i, NIH) using stereological methods.

### 2.6 Mitochondrial respiration

Respiration was assessed in isolated mitochondria from *n* = 8–9 male mice per genotype (mean age 13 weeks). Basal and ADP-stimulated respiration was measured by a Clark-type electrode using the Mitocell S200A Micro Respiratory system (Strathkelvin Instruments, Motherwell, UK) with glutamate (5 mM), malate (2.5 mM), and Na^+^-pyruvate (5 mM) as substrates. See Supplementary material online for details of mitochondrial preparation.

### 2.7 Detergent-resistance measurements (turbidity assay)

Isolated mitochondria from *n* = 6 female hearts per genotype (age 40 weeks) were placed in suspension at 0.5 mg protein/mL. Absorbance was measured at 540 nm every 10 s for 30 min using a 96 well plate reader (Kinetic microplate reader, Molecular Devices) with and without addition of increasing concentrations of Triton X-100 (0–0.05%, TX-100).^22^

### 2.8 Cine-MRI

Mice were anaesthetized with isoflurane 1.5–2% in oxygen with ECG and respiration monitoring to ensure depth of anaesthesia and homeothermic temperature control. Imaging was carried out on a 9.4 T (400 MHz) MR system (Agilent Technologies) using a quadrature-driven birdcage resonator (Rapid Biomedical) as previously described.^15^ Global cardiac functional parameters were measured from double-gated, multi-frame short-axis gradient-echo images covering the heart from the base to apex.

### 2.9 Left ventricular haemodynamics

*In vivo* haemodynamics were performed in closed-chest freely breathing mice under isoflurane general anaesthesia (1–1.5% in medical oxygen) on a homeothermic blanket at 37°C. A 1.4 F mikro-tip catheter (Millar, Houston, USA) was advanced via the right carotid artery, across the aortic valve, and into the LV. The jugular vein was cannulated with stretched polyethylene tubing for administration of dobutamine at 16 ng/g body weight/min to provide maximal β-adrenergic stimulation. Measurements were taken after at least 15 min equilibration using a Powerlab 4SP (ADInstruments, UK) and analysed using Chart Pro software (v5.5.5).

### 2.10 *Ex vivo* model of ischaemia–reperfusion

Female mice from *n* = 10 per genotype (age 21 weeks) were anesthetized with sodium pentobarbital (55 mg/kg I.P.) and heparin (300 IU), hearts were rapidly excised, cannulated, and perfused in Langendorff mode. Hearts were allowed to equilibrate for 15 min, followed by 20 min global no-flow ischaemia, and 30 min reperfusion with function measured throughout via intraventricular balloon. Hearts were perfused for a further 30 min to allow sufficient wash-out of cellular dehydrogenases before staining with triphenyltetrazolium chloride (TTC). Slices were imaged, weighed and then analysed using Image J software to determine infarct size as a percentage of LV.

### 2.11 *In vivo* model of ischaemia–reperfusion injury

Injury was induced in female mice aged 24 weeks under isoflurane anaesthesia and buprenorphine analgesia (1 mg/Kg subcutaneously) via occlusion of the left anterior descending coronary artery for 45 min as described previously.^15^ After 24 h of reperfusion, mice were anaesthetized with intraperitoneal sodium pentobarbital (55 mg/kg) and heparin (300 IU) and the hearts excised for histological analysis of area-at-risk and infarct size using TTC staining as previously described.^23^ A total of 17/24 WT and 18/23 MtCK-OE mice completed the protocol with two WT and one MtCK-OE excluded because the area-at-risk was too small (<20%). This did not affect the outcome of this study.

### 2.12 mPTP assay in isolated cardiomyocytes

Cardiomyocytes were isolated from *n* = 6 female mice per genotype (aged 18–25 weeks) via gravity-driven retrograde perfusion of liberase in Langendorff mode. Cells were plated onto pre-laminated dishes and left at 37°C overnight before commencing experiments. Cardiomyocytes were loaded with tetramethylrhodamine methyl ester (TMRM), which selectively localizes to the mitochondria, and visualized using confocal fluorescence microscopy as previously described.^24^ Photo activation at 543 nm (5% power) generates reactive oxygen species, initiating mPTP opening, and dequenching of the TMRM signal, which is observed as an increase in fluorescence. The known mPTP inhibitor, 0.5 µM cyclosporin A, was used as a positive control.

### 2.13 Mitochondrial swelling assay

A calcium-induced mitochondria swelling assay was used to measure mPTP opening in isolated cardiac mitochondria from *n* = 6 WT and *n* = 5 MtCK-OE female hearts.^25^ Change in optical density at 540 nm was measured for 20 min using a spectrophotometer (Vmax, Molecular Devices, California, USA), with cyclosporin A (1.0 μM) used as a positive control for inhibition of mPTP opening.

### 2.14 Statistical analysis

All experiments were analysed blind to genotype. Data are expressed as mean ± SEM unless otherwise stated. Statistical comparison between two groups at a single time point was by Student’s *t*-test and between three groups or more by one-way ANOVA. A two-way repeated measure (mixed model) ANOVA with Bonferroni correction for multiple comparisons was used to statistically compare groups at multiple time points. Differences were considered significant when *P *<* *0.05.

## 3. Results

### 3.1 Validation of transgenic model

**Figure 1:**
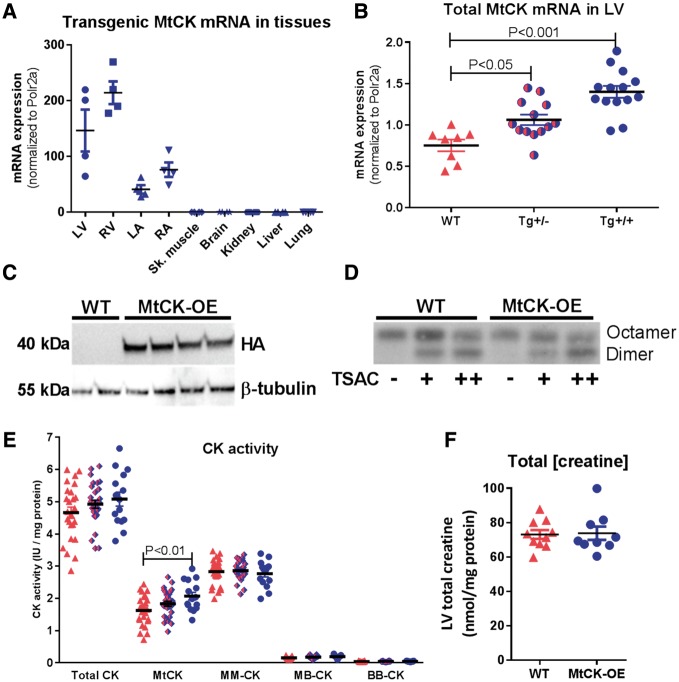
Model validation at mRNA, protein, and enzymatic activity levels. (*A*) Transgenic MtCK mRNA expression in a panel of tissues from MtCK over-expressing mice indicating cardiac specificity for ventricles (LV, RV) and atria (LA, RA) (*n* = 2 M/2 F per tissue). (*B*) Quantitative RT-PCR for total MtCK transcript levels in LV illustrating gene dosing in wild-type (WT, *n* = 5 M/3 F), hemizygous (Tg^+/-^, *n* = 9 M/4 F), and homozygous mice (Tg^+/+^, *n* = 6 M/8 F). (*C*) Western blot showing detection of transgenic protein via haemagglutinin tag in LV tissue from Tg^+/+^ (*n* = 4 male), but not WT mice. (*D*) Incubation of mitochondrial fraction with transition site analogue complex (TSAC) to convert MtCK from octameric to dimeric form, suggesting that under normal conditions all detectable MtCK is in the octameric form in both WT and Tg mice (representative blot from *n* = 3 repeats). (*E*) Activity of the mitochondrial CK isoenzyme is significantly elevated in a gene dose-dependent fashion, with no change in other CK isoforms (shown in order WT *n* = 25; Tg^+/-^*n* = 28; Tg^+/+^*n* = 15). (*F*) LV total creatine levels are not significantly altered by over-expression of MtCK (WT *n* = 10; Tg^+/+^*n* = 9). Data are represented as mean ± SEM.

Offspring produced by hemizygous breeding produced expected Mendelian ratios (WT 72 pups, Tg^+/^^-^ 153, Tg^+/+^ 68; Chi squared = 0.686, two-tailed *P* = 0.71). Development was normal with no significant difference in body weight growth curves over the 11 week follow-up (see Supplementary material online, *Figure S3*). Expression of transgenic mRNA was confined to the four chambers of the heart and was absent in all other tissues samples (*Figure 1A*). A gene-dose effect was demonstrated in left ventricle with homozygous mice showing twice the mRNA expression levels of hemizygotes (*Figure 1B*). There was no change in expression of endogenous mRNA (see Supplementary material online, *Figure S2C*). Expression of transgenic protein was confirmed via detection of the haemagglutinin tag in LV of transgenic animals only (*Figure 1C*). MtCK commonly exists as octamers, but can also form less active dimers.^9^ Mitochondrial fractions were incubated with increasing concentrations of transition site analogue complex (TSAC), which promotes the formation of dimers. In the absence of TSAC, all detectable MtCK was in the octameric state for both WT and Tg hearts, while addition of TSAC resulted in dimer formation regardless of genotype (*Figure 1D*). Transgenic mice exhibited a gene-dose dependent increase in MtCK enzymatic activity, which was 27% higher in homozygote hearts compared to WT (one-way ANOVA; *P* < 0.01). Myocardial creatine levels and activity of the other CK isoenzymes were not significantly altered (*Figure 1E* and *F*).

### 3.2 Mitochondrial localization

**Figure 2:**
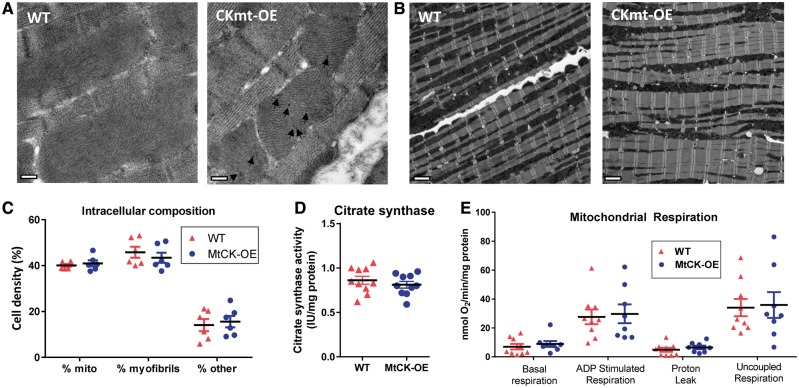
Over-expression of MtCK does not alter mitochondrial phenotype. (*A*) Representative transmission electron microscopy images showing immunogold staining for MtCK–HA was only detectable in LV sections from MtCK-OE mice and was localized to the mitochondria (black arrows; scale bar 200 nm). (*B*) Representative transmission electron microscopy images of male WT and MtCK-OE heart sections (890x magnification, scale bar 2 μm), with quantification of intracellular composition in (*C*), showing that mitochondrial cell density was unchanged (*n* = 6 male). (*D*) Citrate synthase activity as a marker of mitochondrial volume was also unaffected (*n* = 5 M/5 F). (*E*) There were no differences in respiration from isolated mitochondria in the presence of glutamate, malate and pyruvate (*n* = 8–9 male). Data are represented by mean ± SEM.

MtCK is located within the mitochondrial inter-membrane space, where it also plays a structural role, we therefore sought to establish sub-cellular localization and to rule-out potential adverse-effects on the mitochondrial phenotype. Localization of transgenic protein to the mitochondria was confirmed using immunogold staining for the haemogglutinin tag (*Figure 2A*). Scanning electron microscopy also allowed quantification of mitochondrial cell density, which was not altered in MtCK-OE hearts (*Figure 2B* and *C*). This was confirmed by unaltered citrate synthase activity, which is commonly used as a marker for mitochondrial volume (*Figure 2D*). Respiration of isolated mitochondria under basal and ADP-stimulated conditions was not modified by MtCK over-expression (*Figure 2E*). Protein expression of closely associated mitochondrial membrane proteins, ANT, VDAC, and BCL-2 were not significantly altered (see Supplementary material online, *Figure S4*).

### 3.3 ^1^H NMR metabolomics

**Figure 3:**
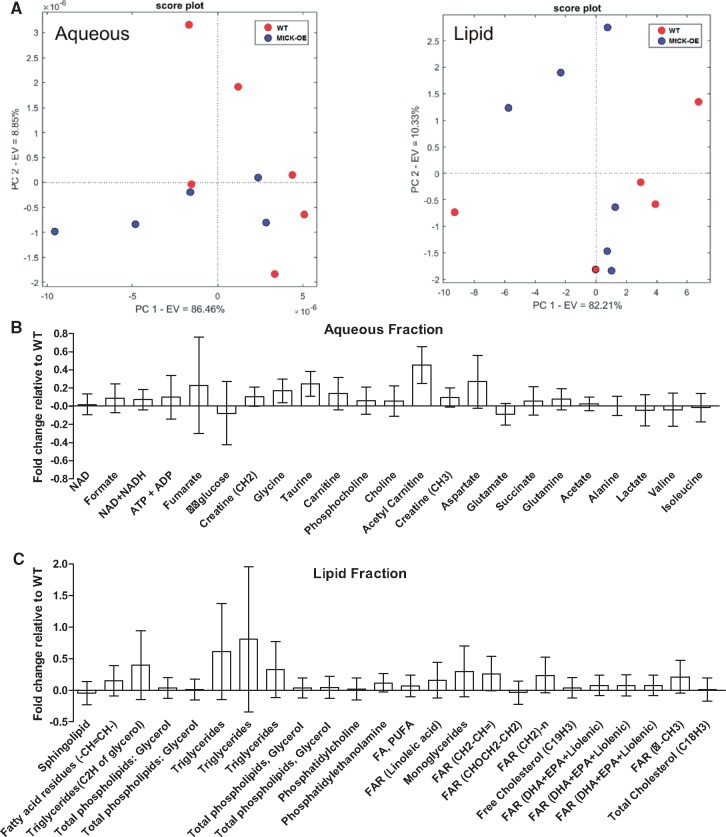
Metabolomic analysis of myocardium by ^1^ H-NMR. (*A*) Unsupervised principal component analysis for aqueous and lipid phases indicates lack of separation based on genotype (*n* = 5/6 females per genotype). Mean fold changes for individual metabolites relative to wild-type hearts for aqueous (*B*) and lipid phases (*C*). Data shows mean ratio ± propagated standard error.

A total of 23 aqueous and 24 lipid metabolites were reliably detected in all samples, however, an unsupervised principal component analysis (PCA) did not detect separation between genotypes for either aqueous or lipid phases (*Figure 3A*). The fold-change for individual metabolites relative to WT hearts are shown in *Figure 3B* and *C* with absolute concentrations provided in Supplementary material online, *Tables S2* and *S3*. Fold changes were small and only acetyl carnitine showed a significant difference between genotypes, with higher levels observed in MtCK-OE hearts (*P* = 0.043). This data suggests that over-expression of Mt-CK does not grossly alter normal cellular metabolism.

### 3.4 Baseline cardiac function

*In vivo* cardiac function was assessed under baseline conditions by cine-MRI and left ventricular catheterization. There were no differences for any parameter of morphology or function between wild-type, hemizygous, and homozygous mice either at baseline or with maximal β-adrenergic stimulation with dobutamine (*Table 1*).

**Table 1:** *In vivo* indices of left ventricular structure and function

	WT	**Tg+/**−	Tg+/+
MRI	*n = 7 (4M/3F)*	*n = 9 (5M/4F)*	*n = 9 (5M/4F)*
End-diastolic volume (µL)	54 ± 15	57 ± 12	58 ± 13
End-systolic volume (µL)	24 ± 9	25 ± 8	26 ± 7
Ejection fraction (%)	55 ± 8	56 ± 5	55 ± 7
Stroke volume (µL)	30 ± 8	32 ± 5	33 ± 8
Heart rate (bpm)	396 ± 78	394 ± 60	462 ± 42
Cardiac index (µL/min/g)	0.52 ± 0.13	0.57 ± 0.10	0.64 ± 0.12
LV mass (mg/g)	3.3 ± 0.6	3.6 ± 0.5	3.5 ± 0.8
Haemodynamics	*n = 10 (5M/5F)*	*n = 10 (5M/5F)*	*n = 10 (5M/5F)*
LV end -systolic pressure (mmHg)	101 ± 6	105 ± 11	104 ± 9
LV end-diastolic pressure (mmHg)	6 ± 3	6 ± 3	6 ± 3
Heart rate (bpm)	444 ± 60	485 ± 91	464 ± 54
*dP*/*dt*_max_ (mmHg/s)	7631 ± 1305	8227 ± 1268	8030 ± 1804
*dP*/*dt*_min_ (mmHg/s)	–4083 ± 533	–4344 ± 495	–4617 ± 915
Tau (ms)	14.4 ± 5.3	17.6 ± 10.6	14.4 ± 5.8
*Stimulated dP*/*dt*_max_ (mmHg/s)	12 006 ± 3014	12 104 ± 1765	10 541 ± 2553
*Stimulated* Heart rate (bpm)	556 ± 60	601 ± 61	570 ± 65
Aortic systolic pressure (mmHg)	96 ± 10	95 ± 15	94 ± 4
Aortic diastolic pressure (mmHg)	65 ± 9	65 ± 16	64 ± 5
Mean aortic pressure (mmHg)	80 ± 9	79 ± 15	78 ± 5

Wild-type (WT), hemizygous (Tg+/−), and homozygous (Tg+/+) mice over-expressing Mt-CK. Stimulated *dP*/*dt* denotes value obtained during maximal stimulation with dobutamine 16 ng/g BWt/min. Data is mean ± SD. No statistically significant differences were observed when transgenic groups were compared with WT using one-way ANOVA followed by Dunnett’s multiple comparison test.

### 3.5 Ischaemia––reperfusion

All subsequent experiments compared homozygous MtCK-OE mice with WT littermates.

**Figure 4:**
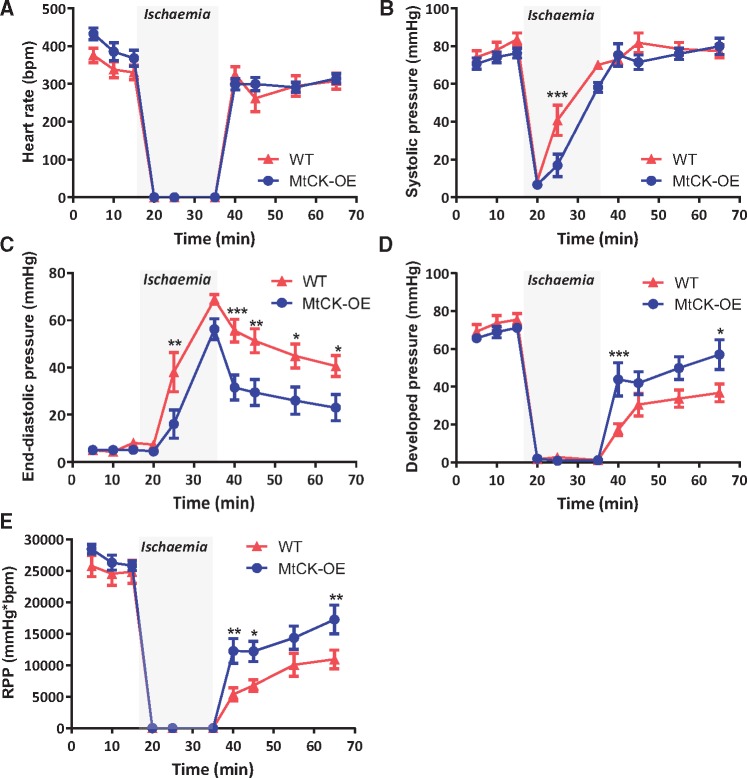
Over-expression of MtCK improves functional recovery following ischaemia–reperfusion *ex vivo*. Hearts were perfused for 15 min at baseline, 20 min no-flow global ischaemia, and 30 min reperfusion. (*A*) Heart rate; (*B*) LV systolic pressure; (*C*) LV end-diastolic pressure; (*D*) developed pressure; and (*E*) rate pressure product, RPP. Data are from *n* = 10 females per genotype and represent mean ± SEM. Between group comparisons by two-way repeated measures ANOVA with Bonferroni post-hoc test. * denotes *P* < 0.05, **P < 0.01, and ***P < 0.001.

*Functional recovery:* Cardiac function was measured in hearts perfused in Langendorff mode for 15 min, followed by 20 min global no-flow ischaemia, and 30 min reperfusion (*Figure 4*). No significant differences were observed during baseline equilibration, however, end-diastolic pressure increased to higher levels in WT hearts during ischaemia and remained higher throughout reperfusion, indicating that MtCK-OE hearts were relatively protected from ischaemic contracture. MtCK-OE hearts also had significantly better systolic function during reperfusion as shown by higher developed pressures and rate pressure product. Heart rates did not differ throughout.

**Figure 5:**
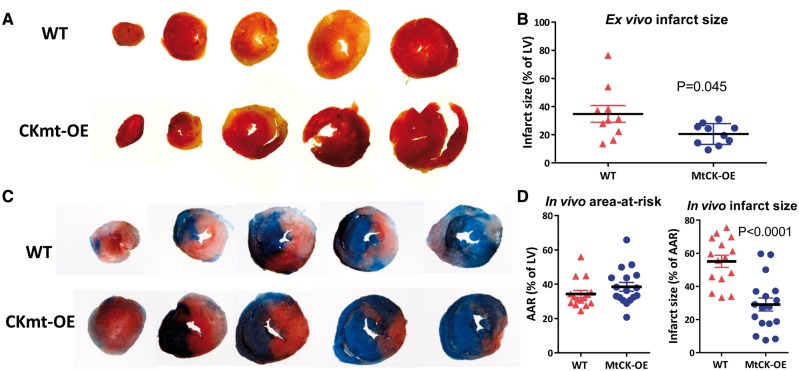
Over-expression of MtCK reduces myocardial injury following ischaemia–reperfusion *ex vivo* and *in vivo*. At the end of experiments shown in *Figure 4*, perfused hearts were sliced and stained with triphenyltetrazolium chloride (TTC), which stains viable tissue red, while necrotic tissue appears pale in colour. (*A*) Shows representative sections spanning apex to base for WT and MtCK-OE hearts, with quantification of infarct size shown in (*B*) and comparison by two-tailed Welch’s *t*-test to reflect unequal variances. The analogous experiment was also performed *in vivo* with 45 min regional ischaemia and 24 h reperfusion. (*C*) Shows representative LV histological sections spanning apex to base. Myocardium that did not experience ischaemia appears blue, all other areas constitute the area-at-risk (AAR), while within this, necrotic tissue (infarct) appears pale or white. Quantification in (*D*) shows no difference in AAR, but a greatly reduced infarct size in hearts from MtCK-OE mice compared to WT mice using a two-tailed Student’s *t*-test (*n* = 17 and *n* = 15 females, respectively). Data are represented as mean ± SEM.

*Reperfusion injury:* The extent of myocardial injury was measured in the same Langendorff perfused hearts after a further 30 min reperfusion (necessary to wash-out dehydrogenases from dead cells for TTC staining^23^). Mean infarct size (% of LV) was 35 ± 6 in WT and 21 ± 2 in MtCK-OE hearts (*P* = 0.045), i.e. 40% less injury in transgenic mice (*Figure 5A* and *B*).

These findings were recapitulated in a separate cohort of mice *in vivo* following 45 min of regional ischaemia and 24 h reperfusion. The percentage of LV exposed to ischaemia is defined as the area-at-risk, and was not different between experimental groups, however, mean infarct size (% of area-at-risk) was 55 ± 4 in WT and 29 ± 4 in MtCK-OE hearts, i.e. 47% less injury in transgenic mice (*Figure 5C* and *D*).

### 3.6 Mechanistic role of mitochondria

**Figure 6:**
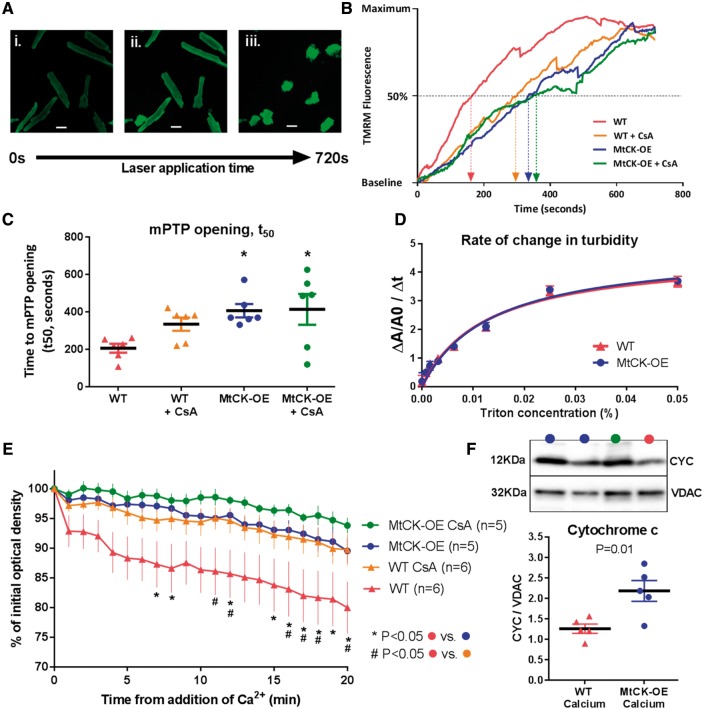
Over-expression of MtCK delays opening of the mitochondrial permeability transition pore (mPTP) *in vitro*. (*A*) Example images of primary cardiomyocytes from mouse heart loaded with fluorescent dye tetramethylrhodamine methyl ester (TMRM). Under baseline conditions TMRM is localized to mitochondria (i), but laser stimulation triggers generation of reactive oxygen species and subsequent mPTP opening as a function of time. This is observed as increased fluorescence due to redistribution of TMRM from mitochondria to the cytosol (ii) and quantified as time to 50% of maximum fluorescence (*t*_50_), which precedes cell death (iii). (*B*) Representative traces of fluorescence against time for cardiomyocytes derived from wild-type (WT) and MtCK over-expressing hearts, with and without the addition of 0.5 μM cyclosporin A (CsA), a known mPTP inhibitor. (*C*) The mean *t*_50_ values from *n* = 6 individual cardiomyocyte isolations per group demonstrate delayed mPTP opening in cells from MtCK-OE compared to WT (* *P* < 0.05), without an additive effect of CsA. (*D*) The turbidity of a suspension of isolated mitochondria changes with addition of detergent (Triton X), with the rate of change, measured by absorbance, reflecting the structural integrity of the membrane. There was no difference in the rate of change over a range of Triton-X concentrations between mitochondria from WT and MtCK-OE hearts (*n* = 6 females per genotype). (*E*) Isolated mitochondria from WT hearts exhibit mitochondrial swelling after addition of 600 μM CaCl_2_, observed as a reduction in optical density over time. Swelling was significantly reduced in the presence of CsA indicating involvement of the mPTP (*n* = 6). Over-expression of MtCK was as effective as CsA alone in preventing swelling, with a small additive effect of CsA, which did not reach statistical significance (*n* = 5). The left ventricle from two male mice were used per experiment and analysis was by two-way ANOVA (*P* = 0.02 for effect of genotype) with Tukey post-hoc test for multiple comparisons. (*F*) At the end of the swelling assay, protein expression of cytochrome c was measured by Western blot and normalized to VDAC. Comparison by unpaired two-way Student’s *t*-test showed significantly more cytochrome c was retained in the MtCK-OE mitochondria consistent with reduced opening of mPTP. Data in all graphs are represented as mean ± SEM.

Myocardial reperfusion injury is intrinsically linked to opening of the mPTP, with subsequent loss of mitochondrial membrane potential and activation of cell death pathways.^13^ To determine the influence of MtCK on this process, cardiomyocytes were isolated from WT and MtCK-OE hearts, and the time to 50% mPTP opening (*t*_50_) was quantified using a fluorescence-based assay (*Figure 6A* and *B*). As expected, pre-incubation with Cyclosporin A (CsA), a known inhibitor of the mPTP, prolonged *t*_50_, although this fell short of statistical significance in WT cells. Nevertheless, in cells from MtCK-OE hearts, *t*_50_ was doubled, indicating a significant delay in mPTP opening (*P* < 0.05). There was no additive effect of pre-incubation with CsA (*Figure 6C*). To confirm these findings we performed a mitochondrial swelling assay, whereby addition of calcium causes mPTP opening and subsequent swelling is detected as a change in optical density. WT mitochondria produced a large reduction in optical density, which was prevented by pre-incubation with CsA confirming that swelling was due to mPTP opening. Mitochondria from MtCK-OE hearts exhibited significantly less swelling compared to WT and were comparable to CsA in ameliorating mPTP opening (*Figure 6E*). At the end of the assay, cytochrome c protein expression was measured in the mitochondrial pellets by Western blot (*Figure 6F*). MtCK-OE mitochondria retained significantly more cytochrome c compared to WT, which is consistent with reduced opening of the mPTP.

MtCK provides contact points between mitochondrial inner and outer membranes, thereby providing structural support to maintain mitochondrial integrity at times of stress.^26^ We therefore sought to determine whether the modest elevation of MtCK in our model was sufficient to benefit from this protective mechanism. Isolated mitochondria from WT and MtCK-OE hearts were exposed to detergent (Triton X) and the extent of membrane disruption was measured as an increase in turbidity (and therefore absorbance) using spectrophotometry. The rate of change in turbidity over a wide range of Triton X concentrations was not modified by genotype (*Figure 6D*), suggesting that additional MtCK-OE did not have a major effect on structural integrity.

## 4. Discussion

In this study, we created a novel murine model over-expressing the mitochondrial isoform of creatine kinase. We confirmed that this did not adversely affect mitochondrial cell density or function and that baseline left ventricular function was normal. MtCK-OE hearts had less ischaemic contracture and improved recovery of contractile function when compared to WT littermates and this was associated with reduced myocardial injury, both *ex vivo* and *in vivo*. In addition to improved energetics via augmented MtCK activity, cardiomyocytes, and mitochondria from over-expressing hearts demonstrated delayed opening of the mPTP.

Our transgenic model was specifically designed to obtain a modest level of transgenic protein expression, since for MtCK to be active, it must reside within the mitochondrial inter-membrane space, where capacity for over-expression is likely to be limited.^11^^,^^14^ The transgene was therefore inserted into a specific validated locus, *Rosa26*,^27^ which avoids issues of random integration and excessive transgene copy number.^28^ We were careful to establish that transgenic protein localized to the mitochondria in the native octameric state, and that mitochondrial cell density and respiratory function were not impaired. The benign nature of MtCK over-expression was confirmed by metabolomic analysis using ^1^ H-NMR, which suggests that normal cellular metabolism was not altered by transgene expression. The only exception was higher acetyl carnitine levels observed in MtCK-OE hearts, which could suggest an abundance of fatty acid supply,^29^ although this was not evident in the lipid phase metabolites. The lack of significant off-target metabolic effects was mirrored by *in vivo* baseline cardiac function, which was shown by MRI and LV haemodynamics to be completely normal.

Clearly, the modest levels of MtCK over-expression also represent a limitation. With activity only 27% above WT levels, this is close to the limits of detection for the protein and activity assays, which had to be powered accordingly. However, this makes the robust level of cardio-protection observed in both I/R models particularly remarkable and it is possible that a less cautious approach could have even greater effect. Furthermore, it is notable that MtCK activity falls by ∼30% in the failing heart,^30^ which our data suggests is sufficient to potentially contribute to the heightened mPTP sensitivity that has been observed in heart failure.^31^

Our findings are in broad agreement with other published reports that have sought to enhance phosphotransfer pathways in the setting of I/R. Most notably, over-expression of the M-CK isoform has been shown to improve recovery of contractile function in Langendorff perfused hearts (n.b. the effect on myocardial injury has not been established).^32^ It is not a foregone conclusion that Mt-CK would also be protective. The CK system is highly compartmentalized with the M-CK isoenzyme existing as a cytosolic dimer localized to the myofibrils, where it catalyses the reverse reaction to regenerate ATP from PCr.^33^ Our study shows that a mitochondrial CK isoenzyme that is functionally coupled to oxidative phosphorylation improves functional recovery post-ischaemia and we present the first evidence for CK protection against myocardial injury, including a demonstration of *in vivo* relevance.

We have previously shown that moderately elevating myocardial creatine levels by over-expression of the creatine transporter (CrT-OE), likewise, improves energetic and functional recovery *ex vivo* and reduces myocardial injury *in vivo*.^15^ Notably, this approach also offered cardio-protection in the presence of co-morbidities such as old age and cardiac hypertrophy.^34^

In both M-CK and CrT over-expression models,^31^P-NMR was used to measure high-energy phosphates during I/R.^15^^,^^32^ M-CK-OE mice had normal PCr at baseline, whereas PCr was elevated in CrT-OE, but regardless, ATP levels were not modified either at baseline or in the response during I/R. Nevertheless, in both strains, recovery of PCr during reperfusion was particularly rapid, attaining higher levels than in wild-type, which could provide energetically favourable conditions for a rapid return to ionic homeostasis, thereby reducing reperfusion injury.^15^^,^^32^ It is a limitation of the current study that we do not have the equivalent^31^P-NMR data for MtCK-OE mice, which means we can only speculate on the likely protective contribution of altered high-energy phosphates or intracellular pH. Likewise, it is a limitation that we have not measured adenine nucleotides or their breakdown products since it is possible that elevated MtCK activity could modify the adenylate energy charge or cellular adenosine release under ischaemic conditions.

Multiple lines of enquiry have implicated MtCK as a modulator of mPTP opening, either directly, or by influencing proteins to which it is functionally coupled, e.g. ANT and VDAC. For example, over-expression of the ubiquitous isoform of MtCK in mouse hepatocytes protected against mPTP opening in response to calcium overload,^16^ but only in the octameric form and in the presence of creatine,^14^ implicating a role for functional channelling of ADP via the ANT.^11^ It should be noted that hepatocytes do not normally express any CK isoforms,^16^ so over-expression may result in an exaggerated response. Our study is the first to confirm delayed mPTP opening with MtCK over-expression in isolated cardiomyocytes and therefore brings physiological relevance to these earlier observations.

The influence of MtCK on the mPTP may be independent of any positive effect on high-energy phosphate metabolism since our assay uses a burst of reactive oxygen species (ROS) to stimulate pore opening, thereby modelling reperfusion, but not the energy deficit inherent with ischaemia. Furthermore, it is likely that MtCK directly influences classical mPTP pathways since there was no additive effect of pre-incubation with the cyclophilin D inhibitor, cyclosporin A. However, we cannot rule out an effect of MtCK on regulators of apoptosis such as bcl-2 and bax, which are located on the mitochondrial outer membrane and may modify sensitivity to mPTP opening.^35^^,^^36^

MtCK has been shown to reduce ROS formation under hyperglycaemic conditions by maintaining ADP cycling and thereby effective coupling with oxidative phosphorylation.^37^ A similar effect has been described for myocardial ANT over-expression, which was associated with reduced infarct size following myocardial infarction.^38^ In addition, MtCK is known to be highly sensitive to inactivation by ROS and in particular peroxynitrite that forms during ischaemia.^39^ In the ischaemic heart, this was shown to promote MtCK transition from octamer to dimer, which contributes to the energetic deficit.^40^ Simply increasing the quantity of functional MtCK octamers may therefore have a protective role, i.e. protection in numbers.

The final mechanism for consideration relates to the structural role of MtCK in maintaining contact sites between the inner and outer mitochondrial membranes, thereby providing mechanical stability.^26^^,^^41^ Mitochondrial-bound hexokinase 2 (HK2) stabilizes these contact sites via interactions with VDAC and ANT,^42^ such that, dissociation of HK2 is associated with increased I/R injury.^43^ Preventing this dissociation and thereby preserving contact sites may underpin the cardio-protective effects of ischaemic preconditioning.^44^^,^^45^ Over-expression of ubiquitous MtCK in the liver has been shown to increase the number of contact sites three-fold, conferring relative resistance to rupture when mitochondria were exposed to detergent.^22^ However, employing the same assay in mitochondria from our MtCK-OE mice, we were unable to detect this effect, perhaps unsurprising, given the high baseline levels of MtCK in heart and the modest level of transgenic protein expression in our model. Any contribution from increased contact points is therefore likely to be minor.

It remains to be seen whether augmenting both enzyme and substrate will have a synergistic effect, but this was behind our decision to create a simple constitutively active model. This will allow us to easily generation double transgenics by crossing MtCK-OE with existing mice that over-express the creatine transporter. We postulate that a double transgenic may rescue the adverse phenotype associated with very high levels of creatine, where CK activity may be limiting in maintaining the optimal ratio of phosphocreatine-to-creatine.^46^

In conclusion, the potential for MtCK to protect against cellular stress has long been recognized, but the proof-of-principle studies at the organ and whole organism level have been missing. Our study provides the first evidence that even a very modest increase in MtCK activity improves post-ischaemic recovery of contractile function in the intact heart, and protects against myocardial injury *in vivo*. This adds to the mounting evidence that augmentation of the CK system is beneficial in I/R and represents a viable therapeutic target. Future work will focus on the identification of small molecules that increase creatine kinase activity for the translation of these findings.

## Supplementary material

Supplementary material is available at *Cardiovascular Research* online.

## Supplementary Material

Online Supplement
