# Supplementary material for: Over-expression of mitochondrial creatine kinase in the murine heart improves functional recovery and protects against injury following ischaemia–reperfusion
Source: Cardiovasc Res. 2018 Mar 2;114(6):858–69. doi: 10.1093/cvr/cvy054 (PMC5909653; doi:10.1093/cvr/cvy054)
Supplement: Online Supplement [file cvy054_online_supplement.docx]

**Online supplement**

**DETAILED METHODS**

**Generation and breeding of transgenic mice**

The open reading frame of mouse sarcomeric Ckmt2 was amplified by PCR from commercially available vector (Geneservice, I.M.A.G.E clone #30296538) using Phusion polymerase (NEB) and cloned into a vector containing the αMHC promoter,^1^ incorporating a *Sal*I and Kozak sequence at the 5’ end/ N-terminus and a C-terminal HA tag/stop sequence/*Hind*III restriction site at the 3’ end (a gift from Dr. Arash Yavari, University of Oxford). αMHC vector and CKMT2-HA fragment were ligated using T4 ligase (NEB), and the ligation product was cloned into *E. coli* by selecting ampicillin-resistant colonies before purifying DNA in the form of Mini and Maxi preps (Qiagen)(Figure s1A). Sequencing of cloned plasmids was outsourced to Source BioScience (Oxford) and were analysed using 4Peaks (A. Griekspoor and Tom Groothuis, mekentosj.com). Consensus sequences were constructed from the individual sequencing reads using Serial Cloner, and subsequently checked for alignment against the predicted sequence.

The αMHC-CKMT2-HA vector was excised at the *Not*I site and cloned in the sense orientation relative to *Rosa26* gene into the exchange vector CB92 at the *Not*I site within the polylinker. Final vector CB92-αMHC-CKMT2 consisted of CKMt2 transgene and the machinery for PhiC31 intergrase mediated cassette exchange at the *Rosa26* locus (Figure s1B).^2, 3^ Sequencing was used to verify cloning junctions and integrity of functional components of PhiC31 integrase. The exchange vector was co-electroporated with an expression cassette for PhiC31 into Acceptor embryonic stem (ES) cells, towards exchanging between the attB sites within the exchange vectors and the attP sites in the *Rosa26* locus (Figure s1C). Cells were selected by gentamicin G418 and resistant colonies were verified for recombination by PCR.

To confirm functionality of the CB92-αMHC-CKMT2 construct prior to transgenesis, it was used *in vitro* towards differentiation of the ES cell clones that would be used for blastocyst implantation. Selected antibiotic-resistant ES cells were plated in the form of 20μl ‘hanging drops’ and differentiated into the cardiomyocyte lineage. Following 5 days, embryoid bodies (EB) were rinsed into culture dishes and supplemented with differentiation media (lacking leukemia inhibitory factor). After 1-2 weeks, EB’s exhibited spontaneous contractile activity. Immunocytochemistry was performed in EB’s fixed using 4% paraformaldehyde (TAAB Labs) for 20 minutes and then permeabilised using 0.5%Triton X-100, followed by blocking of non-specific sites using 10% normal goat serum (Sigma). Primary antibodies were commercially purchased; HA tag: anti-rat (Roche Cat No 11867431001, 1:1500 dilution), αMHC was anti-mouse (Abcam cat No ab50967, dilution 1:200). Fluorescent secondary antibodies were obtained from Molecular probes and were anti-rat Alexa 546 (A-11081; 1:200 dilution) and α-mouse Alexa 488 (A-11001; 1:400 dilution). After counter-staining the nuclei using Hoechst, images were obtained using a Nikon epifluorescent microscope. Representative images are shown in Figure s2A illustrating expression of HA tag and αMHC, confirming that transgene expression was present in, and restricted to, cells which expressed αMHC.

Transgenic mice were generated by microinjecting ES cells into C57BL/6J blastocysts, which were implanted into pseudo-pregnant females and led to chimeric founder mice, which were of mixed C57BL/6J x 129 genetic background. Male chimeric founders were mated with wild–type C57BL/6J females (Envigo, Huntingdon, UK) at 6 weeks. Both founders produced viable offspring, and the first-generation (F1) pups were weaned and genotyped at 3 weeks. Homozygous transgenic (Tg^+/+^) mice were generated by breeding F1 Tg^+/–^ males with F1 Tg^+/–^ females and back-crossed into C57BL/6J genetic background for ten generations.

Pups were genotyped by PCR designed to determine whether a given mouse was carrying two copies of the wild–type allele (wild–type), two copies of the transgenic allele (Tg^+/+^), or one copy of each (Tg^+/–^). A pair of transgene–specific primers were designed (Ckmt2_ex9–10_F x HA_R), which amplify a 158bp fragment at the 3’ end of the transgene, and Primer–BLAST search confirmed that they would not amplify any other sequence in the mouse genome. Since the transgene is integrated at a known locus (ROSA26), a pair of primers binding either side of this site were used (ROSA26_F and ROSA26_R) (see Table S1 for primer sequences). On the wild–type ROSA26 allele, these primers amplify a 508bp fragment. On the transgenic allele, however, the primer binding sites are separated by the >10kbp transgene insert. As a result, the primers anneal to their binding sites, but do not produce a product under standard PCR conditions due to the length of the intervening sequence. Therefore, the 508bp ROSA26 band was used to identify mice which contained at least one copy of the wild–type allele*.* Combining the two pairs of primers into a single PCR produced the bands shown in Figure S2B.

**Genomic DNA extraction and genotyping**

Ear biopsies were digested in 500 µL lysis buffer (100 mM EDTA, 50 mM Tris-HCl (pH 8.0), 1% SDS, 200 mM NaCl) supplemented with 3.3 µL Proteinase K (20 mg.mL). Samples were incubated overnight at 55 ^o^C and vortexed briefly. DNA was further purified by isopropanol/ethanol precipitation. 600 µL isopropanol was added to each lysate, mixed thoroughly by inversion and spun at 13,000 rpm for 10 min at room temperature (RT) in a bench-top micro-centrifuge. The supernatant was discarded, and the pellet washed and re-spun in 500 µL 70% ethanol for 5 min. Supernatants were discarded, and the DNA pellet was air-dried at RT for 30 min. 300 µL T0.1E (10mM Tris, 0.1mM EDTA pH 8.0) was added, and the pellets were allowed to re-suspend overnight at RT. PCR reactions were performed using Immolase DNA polymerase, in 20µl reactions using a SimpliAmp Thermal Cycler (Applies Biosystems by Life Technologies).

**RNA extraction and qRT-PCR**

RNA extraction from mammalian tissues was performed using a combined protocol, incorporating TRIzol extraction followed by spin-column purification. 10-20mg of tissue was homogenized in 500 µl of TRIzol, using a Polytron homogenizer, and centrifuged at 12,000g for 10min at 4°C. Supernatant was transferred to a new tube, incubated for 5min at room temperature, mixed with 100ml of chloroform, incubated for 2min at room temperature, and centrifuged at 12,000g for 15min at 4°C. The aqueous phase was transferred to a new tube, mixed with an equal volume of 70% ethanol, and purified using the Qiagen RNeasy Mini K it according to manufacturer’s instructions (Qiagen). Quantitative RT-PCR (qPCR) was performed using the iScript™ One-Step RT-PCR Kit With SYBR® Green (BioRad). Values for transcript levels were analysed using the 2^-ΔΔCT^ method ^4^ after normalising to reference gene Pol2A.^5^ Primers used in the generation and validation of the MtCK overexpression construct are shown in Table S1.

**Protein extraction and immunoblotting**

Left ventricular samples from WT and MtCK-OE mice were homogenised in ice-cold RIPA buffer (Sigma) supplemented with complete protease and phosphatase inhibitors (Roche) in addition to 1mM DTT, firstly with the PT1200 homogeniser and then with a 19G needle to ensure complete homogenisation. Homogenised samples were centrifuged at 12000rpm for 10 minutes at 4°C and the bicinchoninic acid assay (Pierce Thermo Scientific) performed on the supernatant for protein determination. Samples were normalised for protein concentration and then boiled in Laemmli buffer and reducing solution (NuPAGE, Invitrogen) to allow for direct SDS-PAGE. 25ug of protein were separated on Any kD, Mini-PROTEAN TGX Stain-Free pre cast polyacrylamide gels (Biorad) in 1x Tris/Glycine/SDS running buffer. Proteins were transferred to a nitrocellulose membrane (Biorad) before blocking in 5% dry milk powder with 0.1% Tween 20 in PBS for 1 hour at room temperature. Membranes were incubated with primary antibodies against CKMT (Insight Biotech sc-15169), VDAC (Abcam ab-15895), ANT (Insight Biotech sc-9299) or Bcl-2 (D17C4) #3498 (Cell Signaling Technology, overnight at 4°C followed by 1 hour incubation with appropriate peroxidase-conjugated secondary antibodies. Proteins were detected using the ECL Prime chemiluminescence kit (GE Healthcare) and visualised using the Biorad Chemi Doc MP imaging system. Membranes were stripped and re-probed against GAPDH (Abcam) to allow for normalisation of protein. For cytochrome c protein expression levels, isolated mitochondrial proteins were extracted in RIPA as above, following swelling assays. Total protein was measured as above and 10ug were loaded per well for subsequent analysis using an antibody against cytochrome c (mouse monoclonal [37BA11], Abcam #ab110325). Normalisation was performed using VDAC as a loading control, after stripping and re-probing of the blots.

**Myocardial creatine**

Frozen crushed ventricular tissue was prepared for quantification of creatine by HPLC, and then normalised to protein content using the Lowry method as previously described.^6^

**Creatine kinase activity**

5mg of frozen crushed ventricular tissue was homogenised in 1ml of ice buffer (0.08mM K_2_HPO4, 1mM EGTA, 0.02mM KH_2_PO_4_, and 1mM β-mercaptoethanol. Samples were vortexed and 150µl aliquots removed for determination of protein by the Lowry method. To the remaining sample, a final concentration of 0.1% v/v triton X-100 was added to permeabilise cells. Samples were stored on ice for at least 30minutes to allow precipitation of salts and cellular debris and the supernatant was used for all enzyme activity measurements.

1. **Total creatine kinase activity**

20µl of 1:5 (in ice buffer) diluted sample supernatant was incubated with 1ml of CK-NAC reagent (Thermo Fisher Scientific) at 30°C. After a 3 minute lag time, CK activity was quantified spectrophotometrically by measuring the increase in absorbance at 340nm over 2 minutes as a result of NADH production. The assay was performed in triplicate and results normalised to protein concentration.

1. **Activity of creatine kinase isoenzymes**

Sample supernatant was diluted 1:20 in ice buffer and incubated with 1% CK isoenzyme activator for 10 minutes prior to use. Creatine kinase isoenzymes were separated according to their electrophoretic mobility on an agarose gel, followed by incubation with CK isoenzyme chromogen which allowed for visualisation of the bands. Relative activities of individual CK isoenzymes were quantified by densitometry. All reagents were provided within the SAS-1 CK VIS-12 Isoenzyme kit (Helena Biosciences). Absolute activities for each isoenzyme were calculated by multiplying relative isoenzyme activity by total CK activity.

**Octamer/dimer visualisation on SAS gels**

Dissociation of MTCK octamers into dimers was initiated by the addition of creatine kinase transition site analogue complex (TSAC: 4mM ADP, 5mM MgCl_2_, 20mM Cr and 50mM KNO_3_).^7^ The mitochondrial suspension was diluted 1:20 in ice buffer and incubated with 1% CK isoenzyme activator for 10 minutes prior to use. Creatine kinase isoenzymes were separated according to their electrophoretic mobility on an agarose gel, followed by incubation with CK isoenzyme chromogen which allowed for visualisation of the bands. All reagents were provided within the SAS-1 CK VIS-12 Isoenzyme kit (Helena Biosciences).

**Citrate synthase activity**

50µl of sample supernatant was incubated with 850μl of reaction mixture (0.35mM acetyl-CoA, 0.12mM DTNB) at 25°C. After 3 minutes, 100μl of 1mM oxaloacetate was added into the reaction mixture. Citrate synthase activity was immediately assessed spectrophotometrically by measuring the increase in absorbance at 412nm over 1 minute as a result of 5-thio-2-nitrobenzoate (TNB^2-^) production. The assay was performed in duplicate and results normalised to protein concentration.

**Transmission electron microscopy**

1. **Sample preparation for immunogold staining**

Hearts from WT and MTCK OE mice were extracted under terminal anaesthesia, cannulated via the aorta and perfused with saline to remove any residual blood. Hearts were perfusion fixed based on a published method for the fixation of rodent hearts for electron microscopy.^8, 9^ Briefly, a fixative buffer (0.1M sodium cacodylate, 0.05% calcium chloride, 0.1M sucrose , for an osmolality of 300 mosmols) containing 3% paraformaldehyde and 0.2% glutaraldehyde was perfused via the coronary system to fix the heart. The hearts were sliced into 1-2mm^3^ cubes and immediately submerged into pre-chilled fresh fixative buffer overnight at 4°C. Samples were subsequently rinsed thoroughly in water, and incubated in 0.5% aqueous uranyl acetate overnight at 4°C. Dehydration of samples in increasing ethanol concentrations were performed before the samples were infiltrated with HM20 monostep acrylic resin and polymerised under UV light. 90nm sections were prepared (Diamtome and Leica UC7 ultramicrotome ) blocked and then incubated firstly with rat anti-HA primary antibody (Roche) followed by an anti-rat conjugated to 5nm gold (Abcam, ab81411). Sections were post-stained with uranyl acetate and Reynold’s lead citrate, and imaged in a FEI Tecnai 12 TEM at 120kV.

1. **Mitochondrial cell density**

Samples were collected as described for immunogold staining and prepared as described below. Briefly, a fixative buffer (0.1M sodium cacodylate, 0.05% calcium chloride, 0.1M sucrose , for an osmolality of 300 mosmols) containing 3% paraformaldehyde and 2.5% glutaraldehyde was perfused via the coronary system to fix the heart. The hearts were sliced into 1-2mm^3^ cubes and immediately submerged into fresh fixative buffer for 24 hours at 4°C. Samples were blinded to the operator and 18-20 images were taken per heart at two magnifications, 890x and 1900x, within organised Z-line sections. Images were analysed in Image J (v1.50i, NIH) by overlaying a 9x9 point grid and scoring intracellular organelles beneath each point. The percentage of each cellular component was calculated per grid and data averaged across the 18-20 images per heart. Data was analysed by Student’s t-test.

**Isolation of cardiac mitochondria**

All solutions and equipment were pre-chilled before commencing experiments. Hearts were quickly excised, washed in ice cold phosphate buffered saline (PBS) and the atria and non-myocardial tissue removed. The ventricular tissue was weighed and finely sliced into small sections with a scalpel blade in a petri dish containing Chappel-Perry (CP) buffer (100mM KCl, 50mM Mops, 5.0mM MgSO4, 1.0mM EGTA, 1.0mM ATP, pH 7.4 at 4°C). The heart was transferred to a glass Potter-Elvehjem homogeniser containing 1ml of CP buffer per 100mg of ventricular tissue and gently homogenised using a tapered bottom PTFE pestle. The homogenate was supplemented with 10% 2.5mg/ml trypsin solution and incubated for 10 min at 4°C. The activity of trypsin was diminished with an equal volume of CP buffer containing 2mg/ml fatty acid free bovine serum albumin (BSA). Mitochondria were isolated by differential centrifugation including a slow spin at 900 g to pellet myofibrillar components, followed by centrifugation of the supernatant at 5200 g to pellet the mitochondrial fraction. Mitochondria were washed twice and suspended in KME (100 mM KCl, 50 mM Mops, and 0.5 mM EGTA, pH 7.4), kept on ice and used within 3 h of isolation (protocol adapted from ^10^). Mitochondrial protein concentration was determined by the bicinchoninic acid (BCA) method.

**Mitochondrial Respiration**

Basal respiration was assessed with a Clark-type electrode using the Mitocell S200A Micro Respiratory system (Strathkelvin Instruments, Motherwell, UK). Isolated mitochondria (300µg) were equilibrated in the mitocell chamber in a final volume of 0.3ml respiration medium containing 0.5mM EGTA, 3mM MgCl_2_6H_2_O, 60mM K-lactobionate, 20mM Taurine, 10mM KH_2_PO_4_, 20mM HEPES, 110mM Sucrose and 1g/l fatty acid free BSA, pH7.4 at 25°C. Basal respiration was initiated by the addition of glutamate (5mM), malate (2.5mM) and Na-pyruvate (5mM) as substrates. Sequential additions of 150µM ADP, 2µM oligomycin and 100nM repeated doses of Carbonyl cyanide-*4*-(trifluoromethoxy)phenylhydrazone (FCCP) examined state 3, state 4 and uncoupled respiration, respectively. Non-mitochondrial respiration was determined at the end of the experiment by addition of antimycin A (0.25µg/ml). Quality control was implemented by only including samples with a respiratory control ratio ≥ 3 (RCR; i.e. the ratio of state3: state4).^11^

**^1^H NMR metabolomics**

1. *Tissue harvest and dual-phase extraction of metabolites.* Combined left and right ventricle were removed from 14 week old female mice (n=6 WT; n = 6 Tg) and freeze-clamped using Wollenberger tongs in liquid nitrogen. Frozen tissue samples were crushed, and wet weight recorded. A spatula of heart tissue was dissolved in 2mL each of iced methanol, chloroform, and Millipore water and vortexed. Samples were centrifuged for 1 hour at 3600 rpm at 4°C to separate aqueous, protein, and lipid layers. The aqueous layer was further purified with Chelex 100 through an additional 3600rpm centrifugation step at 4^o^C. The aqueous component was then mixed with 15µL of universal pH indicator solution, and stored at -80^o^C. The lipid layer was placed into a glass scintillation vial, and left to air dry over the course of several hours in fume cupboard at room temperature. Two sample tubes were damaged during transit / storage, which means the final analysis includes only n=5 Tg aqueous phase and n=5 WT lipid phase samples.
2. *NMR analysis of metabolite samples.* Samples were analysed using a vertical-bore, ultra-shielded Bruker 9.4 T (400 MHz) spectrometer with a bbo probe at 298K. Freeze-dried aqueous extracts were re-dissolved in 600µL deuterated water (D_2_O containing 8 g/L NaCl, 0.2 g/L KCl, 1.15 g/L Na_2_HPO_4_, 0.2 g/L KH_2_PO_4_ and 0.0075% w/v trimethylsilyl propanoic acid, TSP) and the pH was adjusted to 7 using 1M HCl or 1M NaOH where necessary. Dried lipid extracts were reconstituted in 600µL deuterated chloroform (CDCl_3_) containing 0.05% v/v tetramethylsilane (TMS). For aqueous samples, a NOESY 1D pulse sequence was used, with 128 scans, 2 dummy scans and 15 ppm sweep width, a repetition time of 5.7s per scan, 90° flip angle and experiment duration of 12.5 minutes. For lipid samples, a ZG pulse sequence was used, with 64 scans, 2 dummy scans and 14 ppm sweep width, a repetition time of 3.5s per scan, 90° flip angle and experiment duration of 3.8 minutes. TopSpin (version 2.1) software was used for data acquisition and for metabolite quantification.  Assignment of metabolites to their respective peaks was carried out based on previously obtained data, confirmed by chemical shift and with reference to published data.^12, 13^ Peak areas were normalised to the TSP or TMS peaks and metabolite concentrations quantified per gram tissue wet weight.
3. *Data Analysis.* Mean metabolite concentrations of transgenic (T) and wild type control (C) groups were calculated and the fold change (T/C) -1 calculated where 0 represents no change while negative or positive values represent a decrease or increase, respectively. The propagated standard error (SEM) of the ratio was calculated using the formula ${SE}_{(T/C)}=(T/C)\sqrt{{({SE}_{T}/T)}^{2}+{({SE}_{C}/C)}^{2}}$, assuming the covariance between the two groups is zero, i.e. C and T are uncorrelated. Between group comparison was by Student’s t-test.

**Detergent-resistance measurements (Turbidity assay)**

Isolated mitochondria from WT and MtCK-OE hearts were suspended at 0.5mg protein/ml in measurement medium (250mM sucrose, 10mM HEPES/KOH pH7.4 and 100μM EGTA at 25°C). Mitochondrial intactness was determined by measuring absorbance at 540nm every 10 seconds for 30 minutes using a 96 well plate reader (Kinetic microplate reader, Molecular Devices) following addition of increasing concentrations of the detergent Triton X-100 (0-0.05%,TX-100). Rate of change in turbidity in response to TX-100 was determined as described previously.^14^

***Ex vivo* model of ischaemia-reperfusion**

Female WT (n=10) and MtCK-OE (n=10) mice were anesthetized with sodium pentobarbital (55 mg/kg I.P.) and heparin (300 IU). Hearts were rapidly excised, cannulated and perfused in Langendorff constant pressure mode at 80mmHg with oxygenated (95% O_2_/5% CO_2_) Krebs-Henseleit buffer pH7.4 at 37°C (mM): NaCl 118, KCl 4.7, MgSO_4_.7H_2_O 1.2, NaHCO_3_ 25, KH_2_PO_4_ 1.2, Glucose 11, CaCl_2_.H_2_O 1.8. LV function was assessed in spontaneously beating hearts using a water-filled intraventricular balloon connected to a pressure transducer (ADInstruments, UK). The left ventricular end-diastolic pressure (LVEDP) was set to 5.2 ± 0.3 mmHg and heart rate (HR) and left ventricular systolic pressure (LVSP) measurements logged. These parameters were used to calculate left ventricular developed pressure (LVDP), (LVSP - LVEDP = LVDP) and rate pressure product (RPP = HR * LVDP). Hearts were stabilised for 15 minutes then subjected to 20 minutes global ischaemia and 60 minutes reperfusion. Function was continually recorded with parameters averaged at 5 min intervals until 30 minutes reperfusion. Hearts were perfused for a further 30minutes to allow sufficient wash out of cellular dehydrogenase enzymes for biochemical analysis with triphenyltetrazolium chloride (TTC). Frozen ventricles were sliced into 1mm sections and incubated with 1% TTC for 25minutes at 37°C, followed by fixation in Shandon Formal FIXX (Thermo Scientific, UK) for at least 2 hours. Slices were imaged, weighed and then analysed using Image J software to determine infarct size as a percentage of LV. Hearts were excluded if they did not attain good baseline *ex vivo* function: criteria RPP >15000 mmHg*bpm and/or LVDP >50 mmHg at the end of the stabilisation period.^15^

***In vivo* model of ischaemia-reperfusion injury**

Injury was induced by occlusion of the left anterior descending (LAD) coronary artery as previously described.^16^ Briefly, female WT (n=24) and MtCK-OE (n=23) mice were anaesthetised with 4% isoflurane in medical oxygen followed by oropharyngeal intubation for mechanical ventilation with 2% isoflurane at 250µl stroke volume and 150 b.p.m (Hugo-Sachs MiniVent type 845, Harvard Apparatus, UK). Absence of the pedal withdrawal reflex confirmed adequate depth of anaesthesia and mice were provided with analgesia (Buprenorphine, 1mg/kg subcutaneous) prior to surgery. A left thoracotomy was performed in the intercostal space between the 4^th^ and 5^th^ ribs, the pericardium removed, and a 6-0 polyethylene suture placed around the LAD. The suture was tied down onto a piece of polyethylene tubing to elicit 45 minutes of ischaemia to a region of the left ventricle. Following ischaemia, the tubing was removed and direct visualisation of blood returning to the ischaemic myocardium confirmed reperfusion. The chest wall was closed and 1ml saline given subcutaneously prior to recovery. Mice were provided with softened chow and supplementary heat until a full recovery was observed. Following 24 hours of reperfusion, mice were anaesthetised with sodium pentobarbital (55 mg/kg I.P.) and heparin (300 IU) and the hearts excised for histological analysis of area-at-risk and infarct size within the left ventricle as previously described using tetrazolium staining.^17^ Mice were excluded if the area-at-risk was smaller than 20% of LV since very small injuries are difficult to assesses accurately. Two WT and one MtCK-OE mouse were excluded on this basis, which did not affect the study outcome.

**Cardiomyocyte isolation**

Cardiomyocytes were isolated from WT (n=5) and MTCK OE (n=5) female mice aged 18-25 weeks. Briefly, mice were anaesthetised with sodium pentobarbital (55 mg/kg I.P.) and heparin (300 IU). Hearts were excised into sterile filtered ice cold Tyrode’s perfusion buffer (pH7.4 (mM): NaCl 130, KCl 5.6, MgCl_2_ 3.5, Hepes 5, Na_2_HPO_4_ 0.4, Glucose 10, Taurine 20) and cannulated via the aorta before gravity-driven retrograde perfusion of the coronary system on a modified Langendorff system at 37°C was performed. Hearts were perfused for 5 minutes with Tyrode’s to ensure adequate clearing of residual blood within the coronaries, before perfusion with digestion buffer for 7-10 minutes (20ml Tyrode’s, 1mg Liberase TM and 0.1mM CaCl_2_) or until flow rate increased by 50% and the heart became pink pale and soft to touch. The heart was cut below the atria, submerged in 5ml of digestion buffer and gently teased apart with tweezers. Tissue was gently triturated for 30 seconds in digestion buffer using a Pasteur pipette and then passed through gauze into a new 14ml round bottom falcon tube. Equal volume of Tyrode’s buffer with 1% BSA was added to terminate Liberase action. The remainder of the tissue from the gauze was triturated in digestion buffer, passed through the gauze and an equal volume of 1% BSA solution added into the same falcon tube. Cells were gently pelleted using a 500 rpm spin for 3 minutes with 1:1 acceleration/deceleration, the supernatant removed and cells re-suspended in increasing concentrations of Ca^2+^ (0.5mM, 1mM). Cells were finally re-suspended in M199 supplemented with 2.5% FBS, 2mM L-carnitine, 5mM taurine, 5mM creatine, 100IU/ml penicillin, 100IU/ml streptomycin and 25µM blebbistatin. This concentration of blebbistatin has been demonstrated to improve cardiomyocyte viability for culture without metabolic off-target effects.^18^ Cells in M199 suspension were plated onto pre-laminated confocal dishes and left at 37°C overnight before commencing confocal experiments.

**mPTP opening assay**

Isolated cardiomyocytes were loaded with fluorescent dye tetramethylrhodamine methyl ester (TMRM) as previously described.^19^ Briefly, cardiomyocytes were loaded with 3µM TMRM (Sigma, T5428) in Hank’s buffered salt solution (HBSS with Ca^2+^, Sigma, H8264) for 15 minutes at 37°C, washed in HBSS and visualised using confocal fluorescence microscopy (Leica TCS SP8 confocal microscope). Lipophillic cation TMRM accumulates selectively into the mitochondria. Photo-activation at 543nm excitation/ 570-590 emission of the white light laser (5% power) generates reactive oxygen species, initiating mPTP opening and dequenching of the TMRM signal, which is observed as an increase in fluorescence and quantitated using Fiji (Image J) Software. Confocal measurements were performed with a 40X oil immersion objective and settings on the microscope were identical to ensure comparability between experiments. The time taken to a 50%-maximal increase in TMRM fluorescence was taken as a measure of mPTP opening. As a positive control, one confocal dish from each individual isolation was supplemented with 0.5µM cyclosporine A (CsA) (Merck, 239835), a known inhibitor of the mPTP.

**Mitochondria swelling assay**

Mitochondria were isolated as per the respiration assay, but with the following modifications. Isolation buffer was sucrose-based (250 mM sucrose, 10 mM HEPES, 1mM EDTA, pH 7.4). Each experiment used mitochondria from two hearts, with each isolated separately before being pooled at the final re-suspension step in 100uL of EDTA-free sucrose buffer. Aliquots of the pooled mitochondria sample were taken for citrate synthase and protein (BCA) assays. Heart mitochondria (1 mg/ml) were suspended in mPTP buffer (250 mM sucrose, 10 mM HEPES, 5 mM KH2PO4, 10 µM EGTA, 10 mM succinate, 1.5 µM rotenone, pH7.4. Mitochondrial swelling was measured by adding 600 µM CaCl_2_ to samples with and without 1μM cyclosporin A, and monitoring the decrease in absorbance at 540nm over 20 minutes using a spectrophotometer (Vmax, Molecular Devices, California, USA).

**Table S1: Primers used for generation of the MtCK overexpression construct (5’- 3’)**

| **Cloning** |  |
| --- | --- |
| Ckmt2_F_SalI/Kozak | 5’GGGGGTCGACACCATGGCCAGTGCCTTCTC3’ |
| Ckmt2_R_HA/stop/HindIII | 5’GGGGAAGCTTCATGCGTAATCTGGAACATCGTATGGGTACTTCCTGCCAAACTGAGG3’ |
| **Genotyping** |  |
| Rosa26_F | 5’ATACCTTTCTGGGAGTTCTCTGCTGC3’ |
| Rosa26_R | 5’GGAGCGGGAGAAATGGATATG3’ |
| Ckmt2_ex9-10_F | 5’TCGGCAGATCAGAGGTTGAG3’ |
| HA_R | 5’TGCGTAATCTGGAACATCGT3’ |
| **qRT-PCR** |  |
| Transgenic sense | 5’TCGGCAGATCAGAGGTTGAG3’ |
| Transgenic anti-sense | 5’TGCGTAATCTGGAACATCGT |
| Endogenous sense | 5’TCGGCAGATCAGAGGTTGAG3’ |
| Endogenous ant-sense | 5’ACCCATAAACTTGGAGGGATAAA3’ |
| Total sense | 5’AGCAAGGATCCACGCTTTTCT3’ |
| Total anti-sense | 5’TCTGCCGATCCGATCTATGTT3’ |
| ApoB sense | 5’CACGTGGGCTCCAGCATT3’ |
| ApoB anti-sense | 5’TCACCAGTCATTTCTGCCTTTG3’ |
| Pol2A sense | 5’TGTGCAGGAAACATGACCGA3’ |
| Pol2A antisense | 5’GAAGCAGACACAGCGCAAAA3’ |

**Table S2: ^1^H NMR aqueous metabolite concentrations**

|  | **WT (*n*=6)**  **µmol/g** | **MtCK-OE (*n*=5)**  **µmol/g** | ***p*** |
| --- | --- | --- | --- |
| NAD | 0.407 ± 0.021 | 0.416 ± 0.042 | 0.87 |
| Formate | 0.222 ± 0.023 | 0.241 ± 0.025 | 0.62 |
| NAD+NADH | 0.418 ± 0.017 | 0.448 ± 0.043 | 0.54 |
| ATP + ADP | 1.20 ± 0.12 | 1.31 ± 0.25 | 0.70 |
| Fumarate | 0.012 ± 0.004 | 0.015 ± 0.005 | 0.67 |
| Alpha-glucose | 0.191 ± 0.056 | 0.177 ± 0.042 | 0.86 |
| Creatine (CH_2_) | 3.95 ± 0.23 | 4.35 ± 0.34 | 0.38 |
| Glycine | 0.279 ± 0.023 | 0.327 ± 0.024 | 0.24 |
| Taurine | 16.3 ± 0.9 | 20.4 ± 1.9 | 0.11 |
| Carnitine | 0.334 ± 0.034 | 0.380 ± 0.045 | 0.47 |
| Phosphocholine | 0.173 ± 0.019 | 0.183 ± 0.017 | 0.72 |
| Choline | 0.073 ± 0.008 | 0.077 ± 0.009 | 0.76 |
| Acetyl carnitine | 0.187 ± 0.015 | 0.272 ± 0.031 | **0.04** |
| Creatine (CH_3_) | 6.17 ± 0.34 | 6.75 ± 0.54 | 0.42 |
| Aspartate | 1.41 ± 0.203 | 1.78 ± 0.32 | 0.38 |
| Glutamine | 3.34 ± 0.35 | 3.04 ± 0.25 | 0.56 |
| Succinate | 0.390 ± 0.046 | 0.412 ± 0.038 | 0.75 |
| Glutamate | 2.70 ± 0.22 | 2.91 ± 0.21 | 0.55 |
| Acetate | 0.463 ± 0.029 | 0.475 ± 0.016 | 0.76 |
| Alanine | 1.15 ± 0.10 | 1.15 ± 0.07 | 0.98 |
| Lactate | 4.35 ± 0.73 | 4.15 ± 0.24 | 0.84 |
| Valine | 0.058 ± 0.010 | 0.056 ± 0.003 | 0.87 |
| Isoleucine | 0.074 ± 0.011 | 0.073 ± 0.003 | 0.94 |

Concentrations were calculated with respect to NMR internal reference standard and normalized to tissue wet weight, i.e. reported as mean ± standard error in µmol/gram wet weight of tissue.

**Table S3: ^1^H NMR lipid metabolite concentration**

|  | **WT (*n*=5)**  **Arbitrary units** | **MtCK-OE (*n*=6)**  **Arbitrary units** | ***p*** |
| --- | --- | --- | --- |
| Sphingolipid | 8.7 ± 1.3 | 8.3 ± 1.0 | 0.84 |
| Fatty acid residues (−CH = CH-) | 794 ± 153 | 914 ± 74 | 0.52 |
| Triglycerides(C2H of glycerol) | 45 ± 15 | 63 ± 13 | 0.43 |
| Total phospholipids (Glycerol (C2H2)) | 67 ± 10 | 69 ± 5 | 0.84 |
| Total phospholipids (Glycerol (C3H2)) | 156 ± 24 | 158 ± 9 | 0.95 |
| Triglycerides (C1H and C3H of glycerol) | 33 ± 14 | 53 ± 11 | 0.33 |
| Triglycerides (C1H and C3H of glycerol) | 31 ± 18 | 56 ± 16 | 0.37 |
| Triglycerides (C1H and C3H of glycerol) | 143 ± 42 | 189 ± 31 | 0.43 |
| Total phospholipids (Glycerol (C3H2)) | 206 ± 30 | 214 ± 12 | 0.81 |
| Total phospholipids (Glycerol (C3H2)) | 85 ± 14 | 88 ± 5 | 0.81 |
| Phosphatidylcholine (−CH2-N-(CH3)3) | 411 ± 67 | 420 ± 25 | 0.90 |
| Phosphatidylethanolamine (−CH2-NH2) | 69 ± 8 | 77 ± 4 | 0.43 |
| FA, PUFA (CH = CH-CH2-(CH = CH-CH2)n, n>1) | 473 ± 73 | 505 ± 25 | 0.69 |
| Fatty acid residues (−CH = CH-CH2-CH = CH-of linoleic acid) | 121 ± 26 | 141 ± 16 | 0.58 |
| Monoglycerides(FA, RH -CH2-CO-O-C2) | 469 ± 129 | 609 ± 89 | 0.43 |
| Fatty acid residues (CH2-CH = ) | 801 ± 154 | 1010 ± 98 | 0.31 |
| Fatty acid residues (COCH2-CH2) | 1100 ± 193 | 1060 ± 84 | 0.86 |
| Fatty acid residues ((CH2-)n) | 6120 ± 1230 | 7590 ± 763 | 0.37 |
| Free Cholesterol (C19H3) | 47 ± 7 | 49 ± 2 | 0.82 |
| Fatty acid residues (ω-CH3 of DHA+EPA+linolenic) | 32 ± 5 | 35 ± 1 | 0.62 |
| Fatty acid residues (ω-CH3 of DHA+EPA+linolenic) | 50 ± 8 | 54 ± 2 | 0.64 |
| Fatty acid residues (ω-CH3 of DHA+EPA+linolenic) | 29 ± 4 | 31 ± 1 | 0.64 |
| Fatty acid residues (ω-CH3) | 1130 ± 205 | 1370 ± 159 | 0.42 |
| Total Cholesterol (C18H3) | 32 ± 6 | 33 ± 2 | 0.95 |

Apparent concentrations were calculated with respect to NMR internal reference standard and normalized to tissue wet weight. Values reported are mean ± standard error in arbitrary units.

**
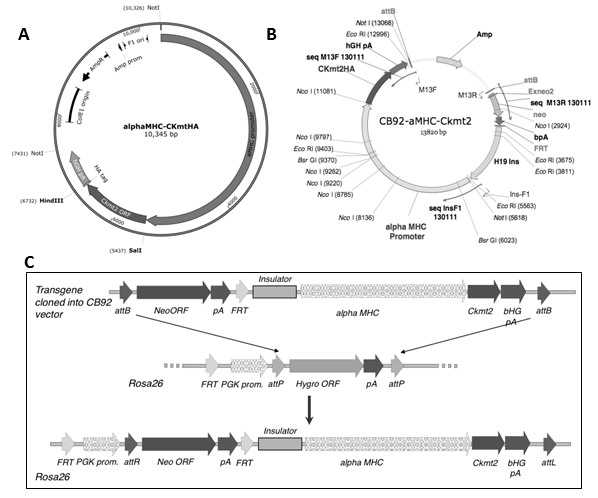
**

**Figure S1 Generation of MTCK2 mice by Rosa26 targeted integration. (A)** αMHC–CKmt–HA construct, with key features and restriction sites used for ligation (*SalI*/*HindIII*). The labelled 7,449bp fragment containing the αMHC promoter, Ckmt2 coding sequence, and polyadenylation signal was excised using *NotI* for cloning into the delivery vector shown in **(B)**; CB92–αMHC–Ckmt2 delivery vector, with recombination sites for PhiC31–mediated integration (attB), neomycin resistance cassette for selection of stably transfected stem cells (neo), and insulator (H19 Ins). Note the *NotI* sites (highlighted yellow) flanking the integrated construct. **(C)** ROSA26 recombination using PhiC31 integrase. The PhiC31 recombination sites (*attB* on the CB92 vector, *attP* on the ROSA26 acceptor site) are modified as a result of integration, generating *attL*/*attR*, which are not substrates for further reactions. This prevents integration of additional copies of the transgene. The neomycin resistance cassette (*NeoORF*, red) confers aminoglycoside resistance for selection of cells in which the construct has integrated downstream of the PGK promoter. The 3’ HA tag attached to the *Ckmt2* coding sequence is not shown. Figures b and c are courtesy of Dr. Ben Davies.

**
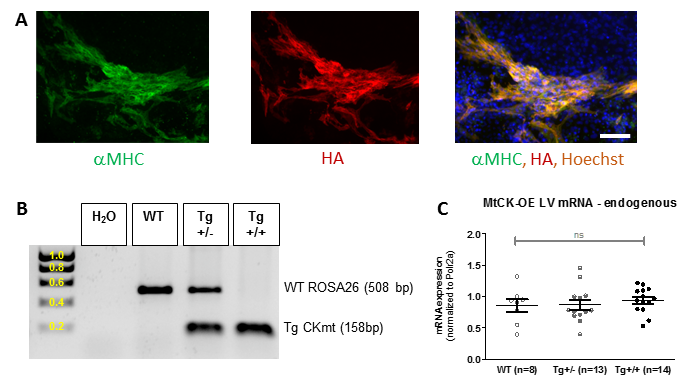
**

**Figure S2 (A)** Immunocytochemistry in differentiated embryoid bodies. Staining against the HA tag (red) and αMHC (green) confirmed that transgene expression was present in, and restricted to, cells which expressed αMHC (merge, yellow). Scale bar, 200μm. **(B)** MtCK–OE transgene zygosity PCR. The ROSA26 primers amplify a 508bp band in mice carrying the wild-type ROSA26 allele (i.e., wild-type and Tg^+/-^ mice), while the CKmt primers amplify a 158bp band in mice carrying the transgene (i.e., Tg^+/-^ and Tg^+/+^ mice). H_2_O, water (no-template) control; wild-type (WT), hemizygote (Tg^+/-^) and homozygote (Tg^+/+^). Ladder markers are in kbp. **(C)** Endogenous expression of CKmt mRNA in WT (n=8), Tg^+/-^ (n=13) and Tg^+/+^ (n=14) hearts.

**Figure S3** Growth curves. Sequential body weight measurements in male (M) and female (F) wild-type mice and hemizygous and homozygous mice overexpressing MtCK in the heart. Data are mean ± SD.

**
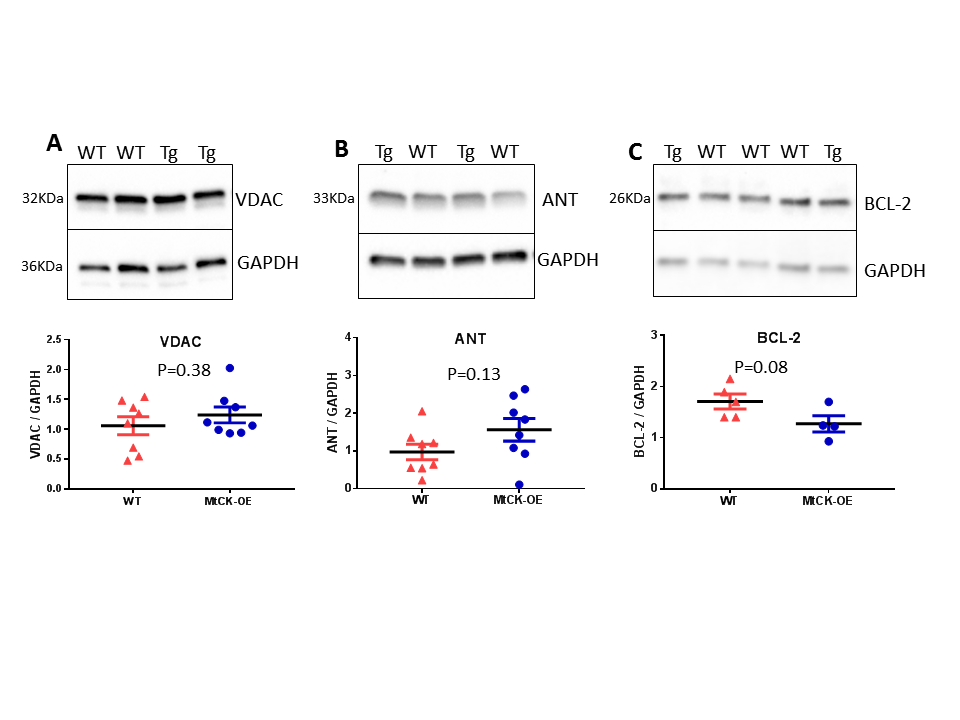
**

**Figure S4** Western blots showing left ventricular expression of associated mitochondrial membrane proteins from wild-type (WT) and mitochondrial creatine kinase over-expressing mice (Tg). Representative blots and densitometry results (mean ± sem with unpaired Student’s t-test) are shown for each. (A) The voltage-dependent anion channel (VDAC); (B) Adenine nucleotide translocase (ANT), both n=10 females per genotype. (C) BCL-2, females n=5 WT and n=4 MtCK-OE.

**Supplementary references**

1. Ng WA, Grupp IL, Subramaniam A, Robbins J. Cardiac myosin heavy chain mRNA expression and myocardial function in the mouse heart. *Circ Res* 1991;**68**:1742-1750.

2. Belteki G, Gertsenstein M, Ow DW, Nagy A. Site-specific cassette exchange and germline transmission with mouse ES cells expressing phiC31 integrase. *Nat Biotechnol* 2003;**21**:321-324.

3. Soriano P. Generalized lacZ expression with the ROSA26 Cre reporter strain. *Nat Genet* 1999;**21**:70-71.

4. Livak KJ, Schmittgen TD. Analysis of relative gene expression data using real-time quantitative PCR and the 2(-Delta Delta C(T)) Method. *Methods* 2001;**25**:402-408.

5. Brattelid T, Winer LH, Levy FO, Liestol K, Sejersted OM, Andersson KB. Reference gene alternatives to Gapdh in rodent and human heart failure gene expression studies. *BMC Mol Biol* 2010;**11**:22.

6. Teerlink T, Hennekes M, Bussemaker J, Groeneveld J. Simultaneous determination of creatine compounds and adenine nucleotides in myocardial tissue by high-performance liquid chromatography. *Anal Biochem* 1993;**214**:278-283.

7. Schlattner U, Wallimann T. Octamers of mitochondrial creatine kinase isoenzymes differ in stability and membrane binding. *J Biol Chem* 2000;**275**:17314-17320.

8. Chen M, Sato PY, Chuprun JK, Peroutka RJ, Otis NJ, Ibetti J, Pan S, Sheu SS, Gao E, Koch WJ. Prodeath signaling of G protein-coupled receptor kinase 2 in cardiac myocytes after ischemic stress occurs via extracellular signal-regulated kinase-dependent heat shock protein 90-mediated mitochondrial targeting. *Circ Res* 2013;**112**:1121-1134.

9. Glyn MC, Ward BJ. Contraction in cardiac endothelial cells contributes to changes in capillary dimensions following ischaemia and reperfusion. *Cardiovasc Res* 2000;**48**:346-356.

10. Rosca MG, Vazquez EJ, Kerner J, Parland W, Chandler MP, Stanley W, Sabbah HN, Hoppel CL. Cardiac mitochondria in heart failure: decrease in respirasomes and oxidative phosphorylation. *Cardiovasc Res* 2008;**80**:30-39.

11. Brand MD, Nicholls DG. Assessing mitochondrial dysfunction in cells. *Biochem J* 2011;**435**:297-312.

12. Jiang CY, Yang KM, Yang L, Miao ZX, Wang YH, Zhu HB. A (1)H NMR-Based Metabonomic Investigation of Time-Related Metabolic Trajectories of the Plasma, Urine and Liver Extracts of Hyperlipidemic Hamsters. *PLoS One* 2013;**8**:e66786.

13. Mayr M, Yusuf S, Weir G, Chung YL, Mayr U, Yin X, Ladroue C, Madhu B, Roberts N, De Souza A, Fredericks S, Stubbs M, Griffiths JR, Jahangiri M, Xu Q, Camm AJ. Combined metabolomic and proteomic analysis of human atrial fibrillation. *J Am Coll Cardiol* 2008;**51**:585-594.

14. Speer O, Back N, Buerklen T, Brdiczka D, Koretsky A, Wallimann T, Eriksson O. Octameric mitochondrial creatine kinase induces and stabilizes contact sites between the inner and outer membrane. *Biochem J* 2005;**385**:445-450.

15. Sutherland FJ, Shattock MJ, Baker KE, Hearse DJ. Mouse isolated perfused heart: Characteristics and cautions. *Clin Exp Pharmacol Physiol* 2003;**30**:867-878.

16. Lygate CA, Bohl S, ten Hove M, Faller KM, Ostrowski PJ, Zervou S, Medway DJ, Aksentijevic D, Sebag-Montefiore L, Wallis J, Clarke K, Watkins H, Schneider JE, Neubauer S. Moderate elevation of intracellular creatine by targeting the creatine transporter protects mice from acute myocardial infarction. *Cardiovasc Res* 2012;**96**:466-475.

17. Bohl S, Medway DJ, Schulz-Menger J, Schneider JE, Neubauer S, Lygate CA. Refined approach for quantification of in vivo ischemia-reperfusion injury in the mouse heart. *Am J Physiol Heart Circ Physiol* 2009;**297**:H2054-2058.

18. Hall AR, Hausenloy DJ. Mitochondrial respiratory inhibition by 2,3-butanedione monoxime (BDM): implications for culturing isolated mouse ventricular cardiomyocytes. *Physiol Rep* 2016;**4**.

19. Hausenloy DJ, Yellon DM, Mani-Babu S, Duchen MR. Preconditioning protects by inhibiting the mitochondrial permeability transition. *Am J Physiol Heart Circ Physiol* 2004;**287**:H841-849.
